# A process evaluation of a family planning, livelihoods and conservation project in Rukiga, Western Uganda

**DOI:** 10.1093/heapol/czae050

**Published:** 2024-11-18

**Authors:** Megan Beare, Richard Muhumuza, Gift Namanya, Susannah H Mayhew

**Affiliations:** London School of Hygiene and Tropical Medicine, Keppel Street, London WC1E 7HT, United Kingdom; Medical Research Council/Uganda Virus Research Institute and London School of Hygiene and Tropical Medicine Uganda Research Unit, Plot 51-59 Nakiwogo Road, Entebbe Uganda; London School of Hygiene and Tropical Medicine, Keppel Street, London WC1E 7HT, United Kingdom; London School of Hygiene and Tropical Medicine, Keppel Street, London WC1E 7HT, United Kingdom

**Keywords:** Integrated programmes, process evaluation, Uganda, family planning, livelihood, conservation, PHE

## Abstract

Although Population–Health–Environment (PHE) approaches have been implemented and studied for several decades, there are limited data on whether, how and why they work. This study provides a process evaluation of the ‘Healthy Wetlands for the Cranes and People of Rukiga, Uganda’ project, implemented by an NGO–local hospital consortium. This programme involved a research design element, testing two delivery modalities to understand the added benefit of integrating conservation, livelihoods and human health interventions, compared to delivering sector support services separately (as is more usual). The process evaluation sought to understand how the programme was implemented, the mechanisms of impact, how it was shaped by the context in which it was delivered and whether there were discernable differences across the two delivery arms. Methods involved key informant interviews with implementing staff and community educators, a review of programme documents and secondary qualitative analysis of interviews and focus groups with community members. The findings include a statistically significant increase in the reach of the programme, in both service delivery and sensitization activities, when the sectors were fully integrated. It appears that this comparative advantage of integration is because of the improved acceptability and motivation among stakeholders, and increased initiative (and agency) taken by community-based peer educators and community members. We argue that the ‘software’ of the programme underpins these mechanisms of impact: trust-based relationships embedded in the system enabled coordinated leadership, supported local staff agency and encouraged motivation.

Key messages

- There are few studies examining whether, how and why cross-sector programmes (e.g. livelihoods, conservation and health) can work.
- This process evaluation shows a statistically significant increase in the reach, in both service delivery and sensitization activities, of the integrated programme compared to providing separate services by sector.
- Comparative advantages of integration are fuelled by improved acceptability and motivation among stakeholders, and the increased initiative and agency of community-based peer educators and community members.
- The mechanisms of impact are underpinned by programme software: trust-based relationships embedded in the system enabled coordinated leadership, supported local staff agency and encouraged motivation.

## Introduction

The concept of connecting programmes and policies, across health and environment sectors is not new. At the programme level, much work has been done over the past 20 years in the so-called ‘PHE’ (Population–Health–Environment) nexus, historically championed by the United States Agency for International Development (USAID) and taken up in various African countries enduring both climate change and rapid population growth (East African Community, 2003). However, despite its longevity, continuing interest in the approach (Sellers, 2019), and five reviews of the field since 2005 (Pielemeier, 2005; Pielemeier *et al*., 2007; Wallace, 2010; Honzak and Oglethorpe, 2011; Yavinsky and Bremner, 2015), there is a lack of consensus on the most effective approaches to programming and governance and a lack of strong empirical evidence on the impact and the processes of successful implementation of such initiatives.

While no standard definition of the PHE approach exists, USAID defines it as ‘population, health and environment interventions that are conceptually linked and operationally coordinated at the field level’ (BALANCED Project T, 2013). Proponents point to a variety of benefits across both health and environment sectors (Yavinsky and Bremner, 2015) and projects have employed a variety of approaches from delivering sector components in parallel, in a coordinated fashion, to fully integrated cross-sector programmes (Oglethorpe *et al*., 2008). It is currently unclear which of these approaches is most effective (BALANCED Project T, 2013) and it has been noted that the classic theories of change used for PHE projects have inadequately explained previous project outcomes; ‘[rules and norms] that influence how theories of change play out in practice are ignored’ (Singleton *et al*., 2019).

The ‘Healthy Wetlands Project’ is an integrated health and livelihoods initiative in Rukiga, south-western Uganda, an area where tensions have arisen between local farming practices and the conservation of local wetlands, affecting wildlife such as the Grey Crowned Crane. It was designed explicitly to address the interconnected health, livelihoods and climate-related challenges that remote communities in this area faced. Integral to the implementation of the project was an evaluation component, which sought to understand how the programme was implemented across the two arms, its mechanisms of impact and how it was shaped by the context in which it was delivered. The results of the process evaluation and their implications for integrative governance and programme approaches are reported in this paper.

## Methods

### Study setting

The ‘Healthy Wetlands for the Cranes and People of Rukiga, Uganda’ project (henceforth ‘Healthy Wetlands Project’) was implemented between 2021 and 2023 by a consortium of the UK-based NGO, the Margaret Pyke Trust (MPT), Rugarama Hospital (a local hospital under the Uganda Protestant Medical Bureau), the Ugandan branch of the International Crane Foundation (ICF) and a UK-based university.

Project sites are located in eight Parishes around the Rushebeya–Kanyabaha wetland, which shrank 33% in the last 35 years, due to the expansion of subsistence agriculture, fuelled by population growth and poverty (Margaret Pyke Trust, 2023). The landscape is hilly with wetlands in the valleys (Bosma *et al*., 2017) and is the habitat of the Grey Crowned Crane, an endangered species whose population declined by 80% in the last 45 years (Mirande and Harris, 2019).

### Study design

The study comprised a cluster quasi-randomized^1^ control trial with two arms, each with four Parishes (eight in total) consisting of multiple villages in each Parish. In the ‘Integrated’ are the interventions which were designed, managed and delivered in a fully integrated manner. This entailed the partners jointly designing the integrated environment/livelihoods and health activities (informed by baseline research findings from the communities involved) and jointly planning the delivery of these activities which were implemented together or with direct reference to one another. Thus, the environment/livelihoods and health partners shared transport, delivered joint activities and made constant reference to all activities. In the ‘Parallel’ are, the same core interventions which were delivered in a coordinated but separate (parallel) manner, whereby environment/livelihoods activities and health activities were implemented separately and involved no integrated messaging (did not cross-reference each other and staff delivering the activities did not, for example, talk about the effects of environmental degradation on health or vice versa). All partners have engaged in the evaluation of the interventions in both arms. This study was approved by the Ethics Committees of the authors’ institutes

**Figure 1.**
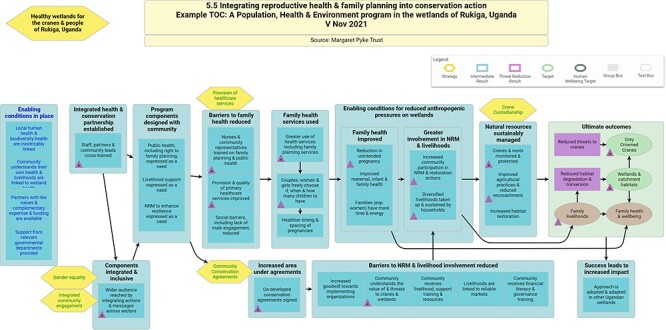
Theory of change for healthy wetlands for cranes and people of Rukiga, Uganda

The intervention activities were informed by a project-wide theory of change, shown in Figure 1. This enabled the project team—which included academics—to identify meaningful points and types of intervention (shown in the hexagonal boxes).

Activities involved service provision and sensitization across conservation, livelihoods and health. Service provision for livelihood supports (seeds and agricultural implements) and conservation equipment was implemented by ICF usually through Community Conservation Groups (CCGs), which were either pre-existing or established with ICF’s support. These groups signed Community Conservation Agreements to facilitate the transfer of support services. Healthcare services were provided through health outreach clinics, including contraceptives and general healthcare. Sensitization involved a wide range of engagement activities, including community meetings held by programme staff and/or peer educators, and the Village Health Teams (VHTs) conducting home visits and engaging in individual conversations with community members.

In parallel sites, all provision and sensitization activities were conducted separately for conservation/livelihoods by ICF and for health by Rugarama Hospital and MPT. In integrated sites, the service and support provision in both conservation/livelihoods and health care made explicit reference to each other and sensitization messaging (at events, by programme staff and peer educators) was explicitly integrated. In integrated sites, peer educators, called ‘Integrated Health and Conservation Mobilizers’, worked on both topics, while in parallel sites these roles were separate. Parallel conservation mobilizers were called ‘Crane Custodians’, whereas those working on health were VHTs and were part of a pre-existing government programme. We refer to them as ‘Peer Educators’ when referring to all categories, ‘Health Mobilizers’ when referring to VHTs and Integrated Mobilizers together, and ‘Conservation Mobilizers’ when referring to Crane Custodians and Integrated Mobilizers together. The activities are summarised in the following table (Table 1).

**Table 1.** Programme activities by intervention arm

Activity	Type	Sector	Programme staff/ Peer educator	Integrated or parallel arm
Community conservation groups	Service provision	Livelihoods	Programme staff	P
Community conservation groups	Service provision	Livelihoods including health	Programme staff	I
Outreach clinics	Service provision	Health	Programme staff	P
Outreach clinics	Service provision	Health, including environment and livelihoods messaging	Programme staff	I
Community meetings	Sensitization	Conservation only	Staff/ Peer educators	P
Community meetings	Sensitization	Health only	Staff/ Peer educators	P
Community meetings	Sensitization	Conservation & health	Staff/ Peer educators	I
Peer sensitization	Sensitization	Conservation only	Peer educators (Crane Custodians)	P
Peer sensitization (incl. home visits)	Sensitization	Health only	Peer educators (VHTs)	P
Peer sensitization (incl. home visits)	Sensitization	Conservation and health	Peer educators (Integrated mobilizers)	I

### Process evaluation framework

The process evaluation framework used in this paper was adapted from the Medical Research Council (MRC) process evaluation framework comprising three main domains: implementation, mechanisms of impact and context (Moore *et al*., 2015).

‘Implementation’ addresses how this project was delivered, its fidelity, its feasibility and its reach. The ‘mechanisms of impact’ include the acceptability of the project, potential mediators, and unanticipated pathways. Finally, ‘context’ includes various factors in the project site and among the project partners that impacted implementation.

This evaluation primarily concerns the comparative differences between the two arms (parallel and integrated) of the intervention, seeking to address the question: ‘Do the implementation, mechanisms of impact, and contextual factors differ depending on the mode of implementation?’

### Data collection methods

The evaluation is based on key informant interviews (KIIs), secondary qualitative sources, routine data, population records and a document review (Table 2).

**Table 2.** Summary of sources

Source type	No. of sources	Additional description
KIIs	10	3 MPT staff, 3 ICF staff, 1 Rugarama Staff, 3 Peer Educators
Secondary qualitative data	7	Transcripts of 3 KIIs with community members from integrated communities.
		Transcripts of 3 FGDs with community members in integrated communities
Routine quantitative programme data	11	
Additional quantitative data	1	
Programme records	12	
Programme evaluations/ reports	11	

Ten KIIs, using a topic guide, were conducted with staff from MPT, ICF, Rugarama Hospital and Peer Educators. These were conducted in English and lasted ∼45 min. Some were conducted by Zoom although all key informants also met in person during a site visit in July 2023. All but one of the interviews were audio-recorded. Informed consent was obtained from all participants.

The secondary qualitative data comprised three KIIs and three focus group discussions (FGDs) conducted with community members as part of the endline impact evaluation (not complete at the time of this study). Informed consent was obtained from all participants.

There were 11 routine data sets, including CCG outputs, community meetings, health centre/outreach, peer educator home visits and conservation outputs. In addition, Rukiga district population data were utilized. It was intended that routine data on family planning uptake at the outreach clinics would be analysed, but due to errors in data collection this was not possible.

The document review comprised 23 documents, including lists of CCG members and peer educators, speech transcripts, programme materials, an external midline review, qualitative baseline report, project reports and staff observation notes.

See Annexe 1 for a detailed description of sources.

### Data analysis

KIIs were transcribed verbatim. These were read and re-read to generate themes that were emerging from the data. A coding framework was developed using a mixture of inductive and deductive approaches. Qualitative data (including secondary data) were analysed using a framework approach and these data were organized and coded in QSR NVivo. Quantitative data were analysed in Microsoft Excel and STATA. Much of the quantitative data did not contain enough observations for standard hypothesis testing, but descriptive analysis is presented here.

Regarding the trustworthiness (credibility, dependability, confirmability and transferability of the results), following the strategies advised by S. Ahmed (Ahmed, 2024), the credibility stems from the triangulation of results between different interviewees, programme documents and quantitative results. The dependability stands on the detailed description of the methods laid out in this paper. Peer debriefing and stakeholder debriefing were performed in pursuit of confirmability. The transferability of the research stands on the detailed discussion of the contextual factors at play.

## Results

### Programme implementation

This section describes the programme reach, fidelity and feasibility in the two arms of the project—integrated and parallel—across three sectors (livelihoods, conservation and health).

### Fidelity of implementation

The livelihood and conservation interventions and the health interventions that were delivered are described below and summarized, by intervention arm, in Table 3. Programme partners reported that everything was implemented as planned in both arms, although there was a delay in commencing health outreach in integrated sites until integrated messaging was finalized.

**Table 3.** Health outreach average numbers

Attendees	Parallel	Integrated	Change in integrated	*P*-value
Total	40.02	43.4	3.35	0.2
Women	30.16	34.4	4.23	0.046^*^
Men	9.866	8.985	−0.88	0.4

* Significant at the 5% level.

#### Livelihood and conservation interventions

The (ICF-led) livelihoods and conservation activities had two components. Firstly, they created CCGs (or supported existing groups). These groups signed agreements with ICF to undertake conservation activities in exchange for support. They received training and equipment for terracing and planting Napier Grass, which prevents water runoff from washing away crops and soil. The groups received seeds and tubers to grow beans and potatoes and were supported to start savings groups to further improve their livelihoods.

Additionally, community members were trained as ‘Crane Custodians’ to provide peer sensitization about cranes and the wetlands both at community meetings and through informal conversations. They were provided with smartphones to document sightings of cranes and crane eggs and were focal points for crane conservation in the community.

#### Health interventions

The health side of the intervention had two components: service delivery and sensitization, for family planning and general health. Health services were delivered through Rugarama Hospital, which held outreach clinics at local health centres (or where no static facilities existed, in places such as churches). Initially, these outreaches provided family planning alone but due to community demand, they broadened their scope to include general medicine, dentistry and optometry.

There were two modes of sensitization: community meetings (including meetings organized in churches and other community meeting places) and peer educators. VHTs were pre-established as voluntary community health workers in Uganda but received additional training from MPT. Their role was informing the community, making referrals to health centres and mobilizing for outreach clinics.

As mentioned, the Crane Custodian and VHT roles were performed by the same people in integrated sites, i.e. they were trained and expected to perform both conservation and health roles, and their numbers reflected this. There were two Crane Custodians and two VHT’s per community in parallel sites, whereas integrated sites each had four Integrated Mobilizers. Integrated Mobilizers were trained to explicitly explain links between conservation, livelihoods and human health. Additionally, at community talks in integrated sites, the relationship between the sectors was emphasized. Project-organized meetings were attended by both ICF and Rugarama Hospital staff but mobilizers also organized meetings themselves.

On their own initiative, Integrated Mobilizers wrote a play and songs incorporating the messages, and formed a drama group to perform at community meetings. An adapted version of the play with conservation messages alone was performed at parallel sites.

### Programme reach

The programme reached people both through providing services (through CCGs or Health outreach clinics) and sensitizing people (through community meetings or peer educators).

#### Community conservation groups

There was one CCG per community, with 40 members on average, and 324 total members, according to programme records. Initially, CCGs averaged 31 members; however, they could admit new members at their discretion, and in 2022 six expanded, with two doubling in size.

Disaggregating by programme arm, while initially the average size was comparable (31.75 in parallel groups and 30.25 in integrated groups), integrated groups added more than twice as many new members on average and integrated groups are now 17% larger (175 total members in integrated vs 149 total members in parallel). All integrated groups opted to add new members, whereas half of parallel groups did not.

Many local government leaders from village and parish levels were CCG members, and key informants reported that these leaders cascaded the conservation activities to their constituents, encouraging or obliging their constituents to adopt these measures too. Therefore, the indirect reach of the project may be much larger. Though beyond the scope of this process evaluation, it will be captured in the endline household survey.

#### Outreach health clinics

According to health outreach data, 223 outreach clinics were held between February 2021 and May 2023, across the eight different sites and 8897 total people attended. Disaggregated by programme arm, 129 outreaches were held in parallel sites and 94 in integrated sites. This difference is primarily because parallel sites started monthly outreaches in February 2021, whereas integrated sites did not have regular outreaches until July 2021 (although one integrated outreach day was held in February) due to a delay in finalizing the integrated sensitization materials. We can therefore only compare arms between July 2021 and June 2023. A paired *t*-test was performed, to evaluate the differences in average attendance between the arms in each month, accounting for seasonal differences.

Integrated sites saw higher average attendance with higher average attendance of women but a slightly smaller average attendance of men (Table 3). The differences in the total number and the number of men is not statistically significant (the decrease in the number of men is less than one person per month), but the increase in the number of women was significant at the 95% confidence level (*P* = 0.046). This indicates that the integrated approach impacts the propensity of women to attend outreach but not men, possibly because of the particular focus was on family planning.

#### Community meetings

There were two sensitization avenues: programme staff and peer educators. Although data are not available for (the many) community meetings held by peer educators, we have data on attendance at staff-run community meetings up to May 2023. There is a discernible difference in reach between the integrated and parallel sites as outlined in Table 4.

**Table 4.** Community meetings attendance

Meeting type	No. of meetings held	Total attendance	Average attendance	*P*-value of difference from integrated
ICF (parallel)	3	423	141	
Rugarama (parallel)	22	2472	113	0.0117^*^
Integrated	16	2773	173	

* Significant at the 5% level.

Given the small sample size for parallel ICF meetings, tests of statistical significance were inappropriate. The sample size for meetings with a health component was also small, but sufficient for non-parametric testing, and a Wilcoxon Signed-Rank Test (paired to account for seasonality) of the difference of average attendance between health talks in parallel sites and the integrated talks gave a *P*-value of  0.0117; a statistically significant result at the 95% confidence level. This indicates that people were more inclined to attend health meetings where conservation topics were also covered.

#### Peer educators

As data on the activities of Crane Custodians and the conservation activities of Integrated Mobilizers was not collected, we cannot compare them. However, the number of home visits made by Health Mobilizers was recorded between November 2022 and June 2023, allowing comparison. As mentioned, the number of Integrated Mobilizers in the integrated sites was double the number of VHTs in parallel sites since, theoretically, their workload was higher given their additional conservation role.

Mobilizers in the integrated sites made nearly double (1.9 times) the home visits than that made by parallel VHTs: 347 compared with 181. The average number of visits per mobilizer was similar, given their greater numbers in integrated sites, though marginally higher in the parallel sites (22.6 visits/mobilizer compared to 21.7 in the integrated sites).

**Figure 2.**
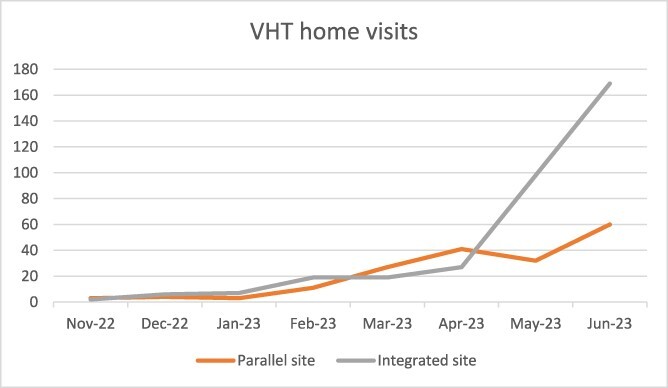
VHT home visits over time, by intervention arm

This difference in activity was not static over the 8 months for which we have data. Activity levels were comparable except during the last 2 months for which we have data: May and June 2023, when visits per mobilizer in the integrated sites rose sharply (Figure 2).

It appears that the reason for this change was a refresher training held in May 2023. Prior to the training, the parallel VHTs were averaging 1.9 visits per person per month, whereas Integrated Mobilizers averaged 0.8 visits. After the additional training, parallel Health Mobilizers made an average of 5.75 visits a month each, whereas Integrated Mobilizers made 8.34 on average.

### Feasibility

Feasibility is cited in the literature as a key benefit of PHE programmes (Yavinsky and Bremner, 2015; Sellers, 2019). We specifically looked at programme coordination, asking ‘did the integration of different sectors create challenges that impeded implementation?’ We also asked ‘did the integration create synergies or opportunities that facilitated implementation?’

#### Programme coordination

Key informants from all partner organizations indicated that the coordination of integrated activities posed initial difficulty, with challenges around the availability of ICF’s field officer, as they did not have additional staff giving them less flexibility.

However, all informants agreed that these challenges had been overcome. After the first year they started holding regular, in-person scheduling meetings. Since then, most informants described the additional managerial burden of integration as negligible, with routine communication and coordination:

*We are now, I think, the best friends. So you know what best friends do? You do everything together*. – KII 6

One senior staff member reported that the complexity of the project made things harder from an organizational point of view, but felt the benefits outweighed the difficulties (KII 5).

Additionally, project partners had to adapt to new ways of working while also learning to trust one another:

*We got used to each other. Because at the beginning ICF staff did not know Rugarama staff, and Rugarama did not know ICF*. – KII 6

Several key informants reflected that though there was a learning process, the challenges ought not to be overstated. All felt that the benefits of integrating the project outweighed the challenges.

#### Synergies and efficiencies

Programme staff reported that integration provided scope for additional synergies and operational efficiency compared to the parallel approach. Integrated community meetings had larger audiences, meaning more people could be sensitized through the same activities. The two organizations conducting field operations (ICF and Rugarama) were additionally able to create efficiencies through coordinating activities. Cohosting community meetings allowed them to carpool, saving fuel and resources. Rugarama also provided ICF with office space in their building, further reducing costs and facilitating communication and coordination.

Overall, while the integration of sectors created both complications and efficiencies, efficiencies outweighed complications. In the short term, parallel implementation was more feasible. However, once the partners mastered coordination, integrating their activities proved more beneficial than working independently.

Table 5 summarised the quanititative results (Table 5).

**Table 5.** Summary of quantitative results

	Parallel	Integrated	Difference
Health outreach average numbers	40.02	43.4	3.35
Average attendance at health community meetings	113	173	60
Average attendance at conservation community meetings	141	173	32
Health Mobilizer home visits	181	347	166

### Mechanisms of impact

Three mechanisms of impact emerged during the evaluation: the acceptability of the interventions, the motivation of stakeholders and community initiative.

#### Acceptability

##### Community

It is difficult to assess the community’s perception of the project, as we relied on secondary data and community members may be reluctant to express criticism. Transcripts of KIIs and FGDs with community members, as well as speeches given by community members, indicate that the intervention was favourably received; all expressed an appreciation for the programme:

*I appreciate [ICF] for coming to this community of ‘X’ parish and I also continue to appreciate the organization of Rugarama* – Community member interview (integrated community)

There was initial scepticism regarding conservation activities, as some community members were concerned that ICF would only help cranes and not them. These fears have been assuaged. It seems the community now credit the cranes with bringing services, and sees their protection as mutually beneficial:

*The crane is the reason why we are getting better services in this community*. – FGD participant (integrated community)

It is not possible to evaluate the comparative acceptability of the two programme arms, as the interviews and focus group transcripts available were only for the integrated sites, as were two of the three speeches. Additionally, the extent to which parallel communities were aware of the connection between ICF and the health outreach is unclear.

We can look at the acceptability of integrating the sectors. The qualitative baseline exercise found the community saw a link between limited access to family planning and the conservation of the wetlands through the pressure on livelihoods that population growth causes. This indicates they were already receptive to this approach (Margaret Pyke Trust, 2019). Staff and mobilizers felt that explicitly linking health and conservation makes the community more receptive to their messages.

*Because we are conserving the crane the people are appreciating, now they’re sending us the doctors and nurses to work with us*. – Integrated Mobilizer, KII 4

An appreciation for the role of ICF in bringing the health outreach to the community was explicitly mentioned by a CCG leader in a speech to programme partners:

*We thank Crane Foundation for having collaborated with Rugarama Hospital where we have received different services like teaching us better methods of family planning* – CCG leader, integrated site.

##### Peer educators

Again, due to the limited sample size it is not possible to compare the favourability of the two arms among the mobilizers with certainty. The mobilizers interviewed liked the project and wanted to continue their roles. Staff who worked with both integrated and parallel mobilizers, reported a perceptible difference in the two groups:

*[Integrated Mobilizers] love it. They feel it’s easy when they’re talking about the wetlands, the cranes, the people, health. They find it easy to relate because this is something that, in real life, it’s happening*. – KII 8

##### Programme staff

Staff in partner organizations expressed a consistent preference for the integrated approach, citing both efficacy and novelty. Multiple staff members said they believed it was the most logical approach:

*Because, our environment is intrinsically linked to our health and vice versa, then that’s what I like about this project*. – KII 5

Field staff mentioned the increased engagement they saw from mobilizers and communities in integrated sites:

*Compared to the parallel sites, the turnout to outreach and community meetings is higher because they understand how family planning interacts with daily living –* MPT Staff Observation Notes

Staff at both field level and managerial level found the integrated project more interesting and exciting to work on:

*When I go for integrated site activities, they are more interesting than when we are in a parallel site*. – KII 7

##### Motivation and enthusiasm

A major driver of the likely impact of integration was the enthusiasm and motivation it engenders in the mobilizers and community.

The motivation of mobilizers affects the reach of the project in two ways: through their direct sensitization of the community and by mobilizing the community to attend community meetings and health outreaches. As one staff member put it:

*The key difference that we’re seeing is the success of these mobilizers*. – KII 5

Some factors that seemed to influence this motivation were evident across categories of mobilizer, some were only evident among integrated and conservation mobilizers and may be due to the particularities of the project, and some were only evident in integrated mobilizers.

Firstly, there is an altruistic motivation. When asked what they liked about their role, both the VHT and the Integrated Mobilizer first mentioned helping others. Integration gives them additional ways to help, and thus increases opportunities for altruistic behaviour.

However, additional factors also seem to affect engagement with the project. Both the Integrated Mobilizer and the Crane Custodian mentioned that their involvement in the programme had increased their standing within their communities:

*People now come to love me so much because of the information I try to give them*. – Crane Custodian, KII 2

In addition, the integrated mobilizer felt his ability to advise people on multiple topics made this especially true:

*As we are integrating knowledge, then people are putting value in us as a people, and love you better* – Integrated Mobilizer, KII 4

As VHTs were already established in their communities, the additional social capital they gain from this project may be less significant.

Furthermore, it seems the novelty of the programme increased enthusiasm among the Integrated Mobilizers, but as the VHTs were already established they felt less excitement:

*The challenge we have made is keeping the VHTs engaged* – KII 8

However, another factor which is unrelated to integration has increased the motivation of mobilizers in certain roles. Crane Custodians and Integrated Mobilizers were given smartphones to send ICF the GPS coordinates of cranes and their nests, which influenced how the community viewed them:

*Some tools that they gave us are part of motivation, like the phones that we use for reporting, communication, whatever. Most of the health mobilizers, they did not have those phones, because this is a developing country, so it became a status*. – Integrated Mobiliser KII 4

Community members seemed to identify the mobilizers as much with the smartphones as they did with the project:

*Sometimes when we are at church, we have those people who were trained from Rugarama, those that were given smartphones…* – Male FGD participant.

##### Community initiative

The community increased the programme’s effectiveness through their own initiative in multiple ways.

As mentioned, integrated mobilizers developed drama and songs to communicate the programme messages to the community, to both draw bigger attendance at meetings and communicate the messages more effectively.

Additionally, the reach of the CCGs was increased through the initiative of their members, firstly, through the decision of the original members to extend membership, secondly, by local leaders involved in CCGs in cascading conservation techniques to their constituents.

### Contextual factors

Several contextual factors emerged which appeared to have an impact: attitudes around cranes; local social and political structures; and the community’s role in identifying solutions.

### Cranes in Rukigan society

Cranes can be a pest, eating crops and causing serious problems as the population are mostly subsistence farmers. Despite this, their image among the community is nuanced. In Uganda, cranes are associated with patriotism; they are the national bird and appear on the flag and money. Several community members mentioned this, and the mobilizers capitalize on this in their messaging:

*Even in our messages we do have that it’s on our National Flag*. – KII 4

People in Rukiga also have traditional beliefs that harming a crane can curse you, causing death or infertility:

*You may die; maybe you cannot produce young ones when they are dying because you touched the crane. –* KII 4

It is worth questioning whether the integration of a family planning project with the conservation of an animal associated with infertility may influence the perception of the project. Staff believed the community were not making that association, but this may be an avenue for future research (KII 10).

### Social and political structures

Social and political structures within the community seem to have had a positive impact on project. Firstly, staff and community members noted that most community members were in some sort of organization:

*Each person has a group that they subscribe to* – Female FGD participant

This facilitated the reach of community meetings, as many of them took place at meetings of local groups or church services, capitalizing on attendees already there, rather than mobilizing from scratch. Furthermore, it may have facilitated the establishment of the CCGs. Some groups already existed in their communities, and ICF helped them register with the government (to ensure legal protection for deposits). Even among groups that ICF created, the members’ prior experience of being in an organization may have helped these groups run smoothly.

Additionally, the project is supported by different levels of local government, due to the cultivation of a relationship with local authorities prior to the programme, by ICF in particular. The District Council even provided land for Napier Grass nurseries, reducing the cost of providing it to the community.

### Community agency

The community’s involvement in developing interventions may also have improved outcomes. As mentioned, large family size was identified by the community as a barrier to conservation during the baseline research, indicating that PHE was the right approach for their challenges. Furthermore, ICF allowed CCGs to select the support they wanted. Though most groups chose the same supports, their involvement ensured the most appropriate intervention at each location, and may have increased buy-in.

## Discussion

Amid growing calls for new kinds of programme partnerships for sustainable development and protection of reproductive rights (Mayhew *et al*., 2020a), little data exist on how these could be developed. This process evaluation has identified factors that shaped the successful implementation and reach of one such innovative cross-sector partnership to provide integrated services.

Two factors seem to underpin the mechanisms of impact: coordination and motivation/enthusiasm. These factors can be understood cohesively as the ‘software’ of a system. Though it has not been investigated in the PHE literature, the software of integration has been investigated in the health service integration literature (Topp *et al*., 2018), including HIV and SRH (Mayhew *et al*., 2020b), family planning and childhood immunization (Hamon *et al*., 2022) and HIV with primary care (Topp *et al*., 2013). A review by Mayhew *et al*. (2021) found ‘hardware factors alone are insufficient for providing effective integrated care’ and sorted software factors into two categories: leadership and governance processes; and the motivation, agency and relationships of frontline providers (Mayhew *et al*., 2020b). These categories map onto the mechanisms of impact found in our study: coordination is a product of the first category, and motivation is an aspect of the second category.

### Benefits of coordinated leadership and shared governance

Synergies and efficiencies are often mentioned in the literature on PHE as a benefit of the approach (Pielemeier *et al*., 2007; Harris *et al*., 2012; BALANCED Project T, 2013; Yavinsky and Bremner, 2015). In our study, strong inter-organizational coordination allowed the project to overcome the complications of an integrated approach, capitalize on synergies, and avoid the pitfalls faced by previous integrated programmes (Wells and McShane, 2004). This study demonstrates that, even if coordination is not initially strong it can be improved.

The consortium structure seems to have enabled close collaboration. The effectiveness of inter-organizational governance has been studied in the field of ‘network governance’, and the work of Provan and Kenis (Provan and Kenis, 2007), may explain this partnership’s success. They contend that partnerships like this, which operate through shared governance, are effective when there is trust at all levels, a small number of partners and a high level of ‘goal consensus’, i.e. everyone works towards the same aims The Healthy Wetlands Project meets all these criteria. Though trust at field level took time to develop, it is now high. Trust at the managerial level appears to be based on MPT’s previous relationship with the other organizations. The number of partners is small, and the interdependence of the sectors in which the partners work means their goals are closely aligned.

### Integration is motivating and should enable staff agency

It seems the motivation, agency and values of staff, peer educators and the community were critical to this programme.

As discussed, motivation seems to have shaped some differences between parallel and integrated arms. The mobilizers’ enthusiasm was the ‘engine’ of sensitization in both arms, but it is unclear which aspect of the project increased motivation: increased scope for altruism, increased social standing because of their additional usefulness to the community, increased social standing because of the smartphones, or the novelty of the project.

The change in the comparative behaviour of the Health Mobilizers in May and June may disentangle these motives. Integrated Mobilizers already had smartphones by then, so even if these did increase motivation, they cannot be the sole driver of behaviour as they cannot explain the later increase in activity. Similarly, the novelty of the project cannot explain this change. While the novelty of the project could explain some differences, the refresher training would not change how interesting the programme was at a conceptual level.

The other potential explanations (altruism and increased social standing from being able to help with more problems) also seem, at first glance, unable to explain this change, as theoretically the refresher training did not give them additional competencies. However, it is possible that they got less out of the initial training, given the amount of additional information they were asked to absorb at that time for their conservation role.

According to staff, integrated trainers were trained in the two sectors back-to-back, and the health training was held last. Additionally, while almost all VHTs were VHTs prior to this project and simply received extra training at the outset (and then refresher training), many Integrated Mobilizers had not performed this role before. It is therefore plausible that Integrated Mobilizers did not feel competent on health topics until after the refresher training, which would explain the uptick, a possibility supported by previous literature which found that perceived self-efficacy increases activity among community health volunteers (CHVs) (Vareilles *et al*., 2017).

Therefore, the question is whether this motivation came from an altruistic desire to use their greater competencies to help others, or because these additional abilities increased their standing in the community.

The evidence on the motivation of CHVs is mixed. Among studies that looked at the relative importance of social standing and altruism, some say social standing has a bigger impact (Banek *et al*., 2015; Vareilles *et al*., 2017), some say they are equally or almost equally important (Brunie *et al*., 2014; Strachan *et al*., 2015), and some say a desire to help is more important (Ludwick *et al*., 2014; Kasteng *et al*., 2016) (Kasteng *et al*., 2016).

While it is difficult to disentangle the effect that the drama had on the programme, the use of drama and music to sensitize Ugandan communities has been studied widely (Frank, 1996; Benge and Kiguli, 2000; Silver, 2001; Barz, 2006; Kendrick and Mutonyi, 2007). The literature has suggested many benefits of the practice, including participation, cultural relevance and sustainability (Silver, 2001). The spontaneous actions of the Integrated Mobilizers, in creating the drama, suggest their substantial motivation and echo the concepts of ‘Theatre for Development’ (Epskamp, 2006) and ‘Theatre of the Oppressed’ (Boal, 1973), both inspired by the work of Brazilian theorist, Paolo Freire (Freire, 1970).

Additionally, we can consider the actions of the CCGs. In adding new members, original CCG members gain nothing, and in fact may have something to lose. If social standing is increased by having something others do not (whether intangible like knowledge or tangible like smartphones or seeds), then the addition of new people to the CCG would decrease the relative social standing of the rest of the group. It therefore appears the motivation for adding new members must be pro-social.

This could be operating through two pathways: either the CCG members increased membership because of benefits they see for the members, or because of benefits they see for the environment. The integrated groups may have felt a greater desire to help others or were more convinced of the importance of conservation. It is difficult to disentangle these motivations. Both may be in play.

It is also worth considering these factors in the context of the hypothesized causal pathways in the PHE literature. Some PHE theories of change do mention increased community engagement as an intermediate outcome (Carr, 2008; Oglethorpe *et al*., 2008; Honzak *et al*., 2012; Balanced Project T, 2013; Singleton *et al*., 2019); however, it seems ‘increased engagement’ is presumed to take the form of increased acceptability. We would argue that this is insufficient and that to maximize the benefits of integration, PHE programmes must allow the community to extend the project on their own terms. Such programme (or systems) flexibility has also been highlighted in literature as important for successful integration (Mayhew *et al*., 2020b). The ultimate outcome should be sustainability beneficial to programmes. While this study did not examine sustainability directly, it seems likely that shared governance and trusted leadership together with engaged communities and motivated staff could collectively contribute to sustained impact over time even if external resources were reduced.

## Limitations

The largest limitation was the lack of data on the actions of peer educators. There were no records of community meetings held without programme staff. Therefore, we have an incomplete understanding of the programme’s reach and the difference in the activity of integrated and parallel mobilizers. Additionally, the lack of data on Crane Custodians activity meant we could not measure their reach, nor see if their enthusiasm differed across programme arms.

The small sample sizes of many variables (such as VHT activity levels) meant the differences between the two arms were not shown to be statistically significant. Furthermore, some contamination between project arms may have occurred, since some community members in parallel sites appeared to know about the integrated partnership between ICF and Rugarama, therefore true differences may be blurred.

As the endline survey (which is expected to provide some information on sustainability) was not completed at the time, and there were errors in the family planning uptake data, it was not possible to incorporate them into this evaluation. In addition, analysis of the sustainability and costing of the programme was beyond our scope.

## Conclusion

We assessed the implementation processes, mechanisms of impact and contextual factors shaping the delivery of livelihoods, conservation and health services in two modalities: sector activities delivered in parallel vs. fully integrated activities.

We found a statistically significant increase in the reach of the programme, in both service delivery and sensitization, when the sectors were fully integrated. It seems this comparative advantage is because of improved acceptability and motivation among stakeholders, and increased initiative (and agency) among community-based peer educators and recipient communities.

We argue that the ‘software’ of the programme underpins these mechanisms of impact and is critical for beneficial programme integration: trust-based relationships embedded in the system enabled coordinated, integrated governance, supported local staff agency and encouraged motivation.

## Data Availability

Routine programme data are available upon request from the implementing partners. Only anonymized and redacted data can be made available in order to protect identifiable information. All interview and focus group discussion data are available and anonymized upon request.

## Footnotes

1. Sites were allocated to intervention or control arms to reduce the proximity of intervention communities to control communities to minimize contamination.

## Reflexivity statement

The author team is strongly inclusive: three women, one man; two Ugandan, one UK and one Irish co-authors; one senior author, one mid-career and two early-career authors.

## Annexe 1

### Sources

Key informant interviews

Ten key informant interviews were conducted, with staff from the Margaret Pyke Trust, the International Crane Foundation and Rugarama Hospital as well as with peer educators.

**Table:**

Organization	Role type
Margaret Pyke Trust	Staff
Margaret Pyke Trust	Staff
Margaret Pyke Trust	Staff
International Crane Foundation	Staff
International Crane Foundation	Staff
International Crane Foundation	Staff
International Crane Foundation	Peer Educator
Rugarama Hospital	Staff
Rugarama Hospital	Peer Educator
Rugarama Hospital & International Crane Foundation	Peer Educator

## Document Review

The documents reviewed were:

- Project records:
  - Signed agreements with Community Conservation Groups (CCGs) 2021
  - Signed agreements with CCGs 2022
  - CCG conservation reports 2023
  - CCG Audit report 2023
  - List of CCG members
  - Materials Used in Community Talks.
  - Darwin Days Delivery Timetables
  - Project Site Maps
  - List of Peer Educators
  - Project Site Names and CCG List
  - Transcripts of two speeches given by CCG leaders to stakeholders during a field visit
  - Transcript of speech given by integrated mobilizer to stakeholders during a field visit
- Project evaluations/reports:
  - External midline evaluation report
  - Report of the qualitative baseline research
  - Project grant application
  - Half year report 2021
  - Annual report 2021
  - Half year report 2022
  - Annual report 2022
  - Annual report 2023
  - Observation Notes

Report of a Socio-Economic Baseline Survey Rukiga Conducted by ICF

- ‘Rukiga project—Alignment with policies and treaties’—Report on the wider policy context by MPT

Programme Routine Data Sets

- CCG Season 1 Livelihood Output Data
- CCG Season 2 Livelihood Output Data
- Community Conservation Groups Money Saved Data
- Community Talks Activity Data
- Health Centre Total Users Not Just on Outreach Days
- Health Outreach Data
- Health Outreach Unmet Need for Contraceptives
- Water Clarity Data 2023
- Crane Census 2033
- Crane Sightings 2021–2023
- Home Visits Made by Health Mobilizers

Non-Programme Data Sets

- Population of Rukiga

Secondary Qualitative Data

I analysed transcripts from three interviews and three focus group discussions conducted as part of pilot of forthcoming endline impact evaluation:

- Transcript of KII with female community member (integrated community)
- Transcript of KII with female community member (integrated community)
- Transcript of KII with male community member (integrated community)
- Transcript of focus group discussion with women (integrated community)
- Transcript of focus group discussion with men (integrated community)
- Transcript of focus group discussion with men (integrated community)
